# Antitumoral activity of allosteric inhibitors of protein kinase CK2

**DOI:** 10.18632/oncotarget.361

**Published:** 2011-12-14

**Authors:** Virginie Moucadel, Renaud Prudent, Céline F. Sautel, Florence Teillet, Caroline Barette, Laurence Lafanechere, Veronique Receveur-Brechot, Claude Cochet

**Affiliations:** ^1^ From INSERM, U1036, Biology of Cancer and Infection, Grenoble, F-38054, France; ^2^ CEA, DSV/iRTSV, Biology of Cancer and Infection, Grenoble, F-38054, France; ^3^ UJF-Grenoble 1, Biology of Cancer and Infection, Grenoble, F-38041, France; ^4^ CEA, iRTSV/CMBA, Grenoble, F-38054, France; ^5^ IMR Laboratory CNRS UPR3243, IMM, F-13402 Marseille cedex 20, France

**Keywords:** Protein-kinase CK2, Inhibitors, Azonaphthalene, Cancer, SAXS

## Abstract

**Introduction:**

Due to its physiological role into promoting cell survival and its dysregulation in most cancer cells, protein kinase CK2 is a relevant physiopathological target for development of chemical inhibitors. We report the discovery of azonaphthalene derivatives, as a new family of highly specific CK2 inhibitors. First, we demonstrated that CK2 inhibition (IC_50_= 0.4 μM) was highly specific, reversible and non ATP-competitive. Small Angle X-ray Scattering experiments showed that this inhibition was due to large conformational change of CK2α upon binding of these inhibitors. We showed that several compounds of the family were cell-potent CK2 inhibitors promoting cell cycle arrest of human glioblastoma U373 cells. Finally, *in vitro* and *in vivo* assays showed that these compounds could decrease U373 cell tumor mass by 83% emphasizing their efficacy against these apoptosis-resistant tumors. In contrast, Azonaphthalene derivatives inactive on CK2 activity showed no effect in colony formation and tumor regression assays. These findings illustrate the emergence of nonclassical CK2 inhibitors and provide exciting opportunities for the development of novel allosteric CK2 inhibitors.

**Background:**

CK2 is an emerging therapeutic target and ATP-competitive inhibitors have been identified. CK2 is endowed with specific structural features providing alternative strategies for inhibition.

**Results:**

Azonaphthalene compounds are allosteric CK2 inhibitors showing antitumor activity.

**Conclusion:**

CK2 may be targeted allosterically.

**Significance:**

These inhibitors provide a foundation for a new paradigm for specific CK2 inhibition.

## INTRODUCTION

Protein kinase CK2 plays critical roles in cell growth and differentiation, apoptosis and oncogenic transformation [1, 2]. Aberrant CK2 kinase expression was associated with unfavorable prognostic markers in prostate cancer [3] and in acute myeloid leukemia [4] implicating CK2 in tumor formation and recurrence. Its dysregulation in many other cancers together with its dual function in promoting cell growth and in suppression of apoptosis may be particularly relevant to its oncogenic potential [5]. Further relevance for its role in cancer is indicated by its mediation of resistance to chemotherapy-induced apoptosis [6]. Involvement of CK2 in other diseases such as glomerulonephritis [7] or viral infections (HIV, CMV, HPV, PV…) has been reported [8]. Based on these observations, CK2 is now considered to be a relevant physiopathological target amenable to therapeutic intervention therefore supporting the identification and the characterization of chemical inhibitors [9, 10]. Extensive efforts have been focused on developing CK2 inhibitors and several active, and in certain cases selective, ATP site-directed compounds have been identified [11]. These include condensed polyphenolic compounds such as emodin and derivatives of hydroxycoumarins (3-carboxy-4(1H)-quinolone), the indoloquinazoline derivative (IQA), tetrabromocinnamic acid (TBCA), 4,5,6,7-tetrabromo-1-benzotriazole (TBB) and pyrazolo[1,5-a][1,3,5]triazine derivatives [12]. Recently, our group and others reported the identification of very efficient CK2 inhibitors with good *in vivo* potency [13-15].

Beside ATP-competitive inhibitors binding to the canonical ATP-site, small molecules targeting different surfaces of kinases [16-18], including CK2 [19, 20] have been identified. Some of them bind to the hydrophobic CK2ß-binding cavity on CK2α, possibly inducing an inactive conformation [21]. Indeed, an inactive conformation of the catalytic CK2α subunit was recently reported [22]. In this CK2α structure, it has been suggested that the binding of small molecules to the CK2ß-docking site have an inhibitory impact on CK2α by promoting its inactive conformation [21, 22]. Altogether, these observations suggest the existence on CK2 of different exosites distinct from the catalytic cavity that can be targeted by small molecules to achieve functional effects [19].

Using an automated screening, we have identified azonaphthalene derivative compounds as new highly potent CK2 inhibitors.

We report that azonaphthalene derivatives are specific non ATP-competitive CK2 inhibitors. Small Angle X-Ray Scattering analysis showed a major conformational change of the kinase upon inhibitor binding, Furthermore, several compounds of the family are cell-permeable CK2 inhibitors promoting cell cycle arrest of human glioblastoma U373 apoptosis-resistant cells. Finally, we demonstrate that these compounds decrease tumorigenesis *in vitro* and exhibit *in vivo* efficacy in tumor growth assays.

These results show that a relevant allosteric inhibition of CK2 activity can be achieved with non-ATP competitive inhibitors expanding the options to modulate this enzyme.

## RESULTS

### Identification of a new potent CK2 inhibitor scaffold

**Figure 1:**
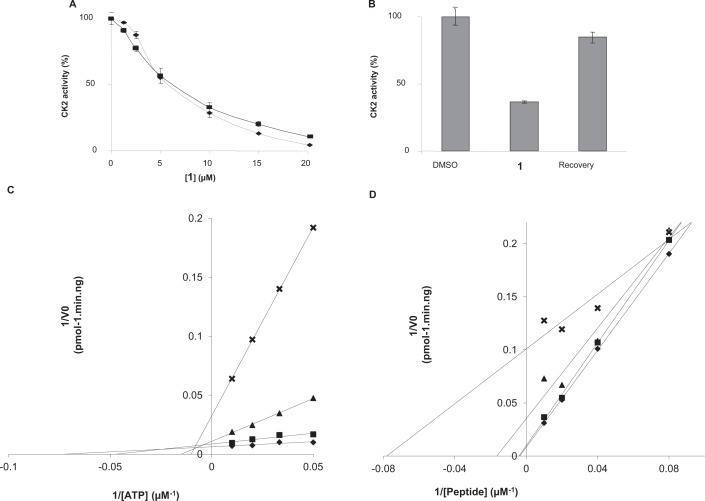
Characterization of compound 1 as a reversible non-competitive CK2 inhibitor **A.** Inhibition of the catalytic activity of recombinant 36ng CK2α (♦) or 60ng CK2 holoenzyme (α_2__2_) (■) by increasing concentrations of compound **1**. **B.** Reversibility of compound **1** inhibition. CK2α (2μg) was incubated with 25μM compound **1** following by a gel-filtration chromatography. CK2 kinase activity in input and flow-through were then assayed. **C** and **D**. Lineweaver–Burk inhibition plots of human recombinant CK2α by compound **1**. CK2 kinase activity was determined as described in the experimental section in the absence (♦) or in the presence of 2.5 (■), 5 (▲) and 7.5 (**X**) μM compound **1** with various concentration of ATP (**C**) or peptide substrate (**D**). The data represent means of experiments run in triplicate with SEM never exceeding 10%.

The 2,860 compounds from the National Cancer Institute Developmental Therapeutics Program small molecule library were screened in an automated luminescence-based *in vitro* kinase assay against the human recombinant CK2 catalytic subunit CK2α as previously published [21]. As a primary screen, CK2 kinase inhibitory activity was determined by measuring the percentage of inhibition at a compound concentration of 15 μM, using TBB and DMSO as positive and negative controls respectively. A secondary screen performed at a compound concentration of 1.5 μM allowed the isolation of 11 hits. Hit validation was performed at concentrations of 1.5 μM using standard radiometric kinase assay with high ATP concentrations (100 μM, *K*_m_ for ATP of recombinant CK2: 25 μM) to enhance the probability of isolating non-ATP competitive compounds. This led to the characterization of compound 1 as micromolar CK2 inhibitor (Figure S1). Compound 1 displays an IC_50_~ 5 μM on CK2α and is equally potent on CK2α alone or complexed with its regulatory CK2β subunit (Figure 1A).

Given that this active compound is hydrophobic, planar and rigid with peripherical polar groups, we investigated whether it could behave as promiscuous inhibitors [23], acting via an aggregation mechanism [24, 25]. Since aggregate-forming inhibitors often display steep dose-response curves and high Hill coefficients [26, 27], we performed dose-response curves for CK2 inhibition. It was observed that compound 1 displayed standard dose response curves with Hill slopes ranging between 1.1 to 1.3 indicating that the compound likely binds its target enzyme through a classical 1:1 inhibition mechanism. Moreover size-exclusion chromatography experiments indicated that CK2α inhibition by compound 1 is reversible (Figure 1B), a feature that is rarely associated with aggregating compounds. Taken together, this rules out compound 1 as a promiscuous aggregator. To define the mode of CK2 inhibition by compound 1, we performed steady-state kinetic analysis. Lineweaver-Burk inhibition plots showed that inhibition pattern of CK2α by compound 1 was non competitive toward both ATP and peptide substrate (Figure 1C and 1D). Of note, at high peptide substrate concentrations the plots were non-linear suggesting that in the presence of saturating substrate concentrations, the enzyme may be more susceptible to inhibition by compound 1.

### SAXS analysis of CK2α-1 complex

**Figure 2:**
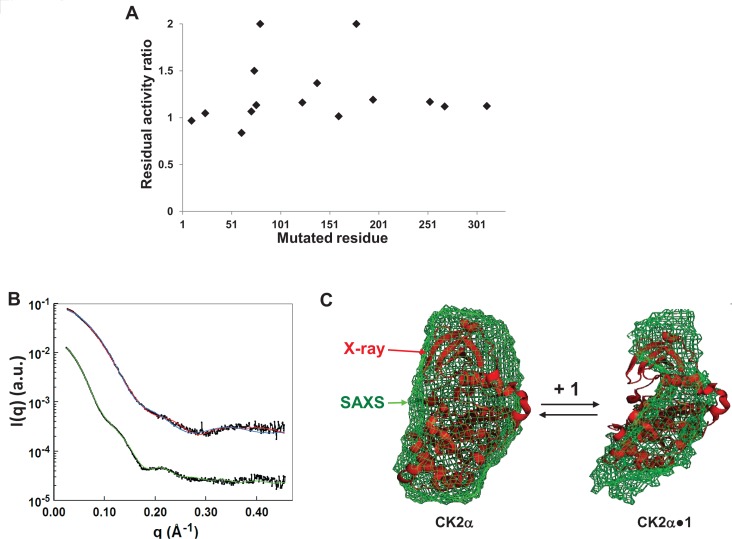
Structure of CK2α and CK2α-1 complex as determined using SAXS Ab initio shape restoration **A.** Inhibitory effect of compound **1** on WT and a panel of mutants with the following residues mutated to alanine (10, 24, 61, 71, 74, 76, 80, 123, 138, 160, 178, 195, 253, 268, 308, 311). Residual activity ratio is defined as the normalized activity of mutant CK2α divided by the normalized activity of wild-type CK2 in presence of 5μM compound **1**. **B**. Experimental scattering spectrum of CK2α (black line, above) and of CK2α-**1** complex (black line, below), and theorical spectra of CK2α calculated by CRYSOL using the crystal structure of CK2α (PBD ID 1PJK) as template (red line), and of the shape calculated by GASBOR for CK2α (blue line) and CK2α-**1** complex (green line). The curves have been artificially shifted for better lisibility. **C.** Typical calculated shape obtained by GASBOR (green envelope) of CK2α (left panel) and CK2α-**1** complex (right panel), superimposed with the atomic structures of CK2α (PDB ID 1PJK). The atomic structure is in red ribbon representation.

To determine the binding site of compound 1, we tested its effect on a series of CK2α mutants (Figure 2A). This revealed that CK2α mutated on K78A, R80A or L178A were more resistant to compound 1 inhibition, suggesting that these residues located on helix αC and on the activation segment are part of the compound 1 binding site. To get better insights into the molecular interactions between CK2α and compound 1, we tried to obtain X-ray crystallographic co-structure of CK2α-1 complex. However, despite several crystallization screening campaigns, we were unable to get diffracting crystal containing CK2α complexed with compound 1.

Therefore, we performed Small Angle X-Ray Scattering (SAXS) experiments on rhCK2α^∆C^(1-335) and rhCK2α^∆C^(1-335)-1 complex (Figure 2B). SAXS is indeed a very appropriate tool to assess the structural properties (dimensions, flexibility and overall shape) of proteins in solution. The Guinier law allows the scattered intensities to be approximated, at low scattering angles to determine the radius of gyration (*R*_G_). When applied to CK2α, *R*_G_ values calculated for different protein concentrations revealed that upon compound 1 binding, *R*_G_ (extrapolated to zero concentration) increases from 32.8±1.2 Å to 35.6±1.1 Å at 20°C and the corresponding Maximal diameter (Dmax) increases from 68±3 Å to 96±4 Å. As probed by kinase assay, compound 1 inhibition is reversible and Kratky plot of CK2α-1 SAXS data exhibits a bell-shaped profile typical of globular protein. Thus, a denaturation of CK2α by compound 1 can be ruled out. The overall shape of rhCK2α^∆C^(1-335) and rhCK2α^∆C^(1-335)-1 complex were calculated *ab initio* using the program GASBOR (Figure 2C). Different runs gave similar shapes. Averaged calculated shape of rhCK2α^∆C^(1-335)-1 complex superimposed with the X-ray structure of rhCK2α^∆C^(1-335) (PDB ID 1PJK) shows that CK2α undergoes a conformational change, leading to a distorted shape. In this conformation, CK2α could be inactive due to non-optimal spatial arrangement of its catalytic site. Alternatively, some domain movement may be impaired impeding catalysis.

### Effect of compound 1 on cellular CK2 kinase activity

**Figure 3:**
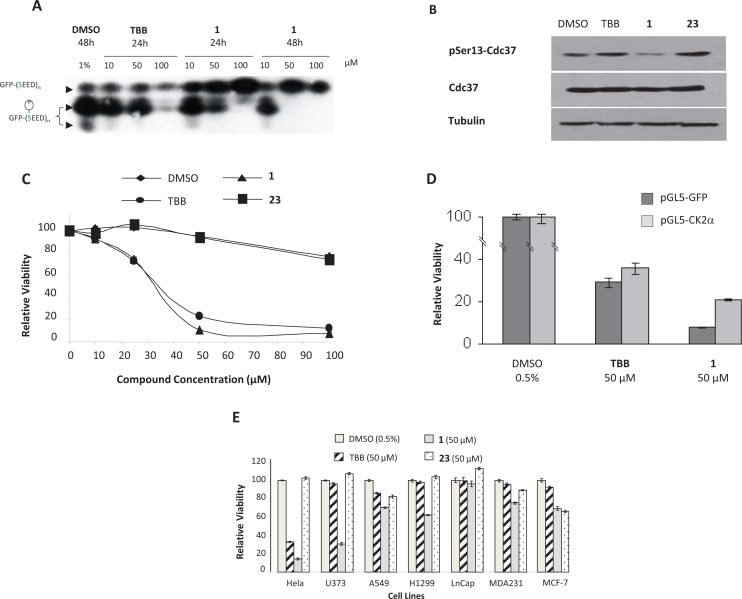
Compound 1 is a cell-potent CK2 inhibitor and decreases cell viability in a CK2 dependent manner **A.** HeLa cells were plated and transfected with the CK2 activity reporter plasmid. One day after, medium was replaced with medium containing increasing amounts of compounds and incubated for 24h or 48h. Then, cells were collected and the reporter phosphorylation status was measured from whole cell extracts. Experiment was repeated 3 times. **B.** U373 cells were plated one day prior inhibitor addition. Twenty four hours after compound addition, cells were collected and phospho-Cdc37, Cdc37 and tubulin levels were measured by immunoblotting. Experiment was repeated twice. **C.** One day after plating, U373 cells were treated with increasing concentrations of TBB, **1**, **23** or DMSO. Two days after, living cells were counted as described. Results are given relative to the luminescence recorded for DMSO. Experiment was done in triplicate and repeated twice. **D**. HeLa cells were transfected with a CK2α or a GFP-expressing plasmid. One day later, medium was replaced with medium containing 0.5% DMSO or 50 μM TBB, **1.** Two days after, living cells were counted as in **C**. Results are given relative to the luminescence recorded for DMSO in each condition. Experiment was done in triplicate and repeated twice. **E.** One day after plating, cells were treated with 50 μM TBB, **1**, **23** or 0.5% DMSO. Two days after, living cells were counted as in **A.** Results are given relative to the luminescence recorded for DMSO in each cell lines. Experiment was done in triplicate and repeated twice.

To evaluate the efficacy of compound 1 to target CK2 into living cells, we used a cellular CK2 activity assay [28]. Compound 1 tested at increasing concentrations for 24 or 48 h was active on cellular CK2 activity (Figure 3A). This was also confirmed by immunoblotting using a phosphospecific antibody recognizing Cdc37 phosphorylated on Ser13 which is specifically targeted by CK2 [29]. Thus, Ser13-Cdc37 phosphorylation status can be used as a surrogate cellular CK2 activity assay [29]. We found that under similar conditions (50 μM, 48h incubation), compounds 1 like TBB, reduced drastically Cdc37 phosphorylation on Ser13. Compound 23, an analogue of compound 1 which is known to be cell-permeable [30] was inactive both on recombinant CK2α and on cellular CK2 activity, (Figure 3B).

### Compound 1 decreases cell viability in a CK2-dependent manner

CK2 inhibitors are known to decrease cell viability. Thus, we measured HeLa cell viability after 48h treatment with increasing amount of TBB or compound 1. Compound 23 was used as negative control. Viability of HeLa cells exposed to compound 1 or TBB showed similar decreased viability (IC_50_ 33 μM and 36 μM respectively) (Figure 3C). To determine whether the reduction of cell viability triggered by compound 1 was mediated by CK2 inhibition, we transfected HeLa cells with a CK2α or a GFP-expressing plasmid. Cells expressing exogenous CK2α displayed a better survival when treated with compound 1 than control cells (Figure 3D). Then, we measured the effect of compound 1 on the viability of different known tumor cell lines (Figure 3E). At a concentration of 50 μM, compound 1 decreased viability of most tested cell lines and was even more efficient than TBB, especially in p53 mutant cell lines like U373 and to a lesser extent H1299 and MDA231. This decreased viability was correlated with a down regulation of CK2 activity attested by Cdc37 Ser13 phosphorylation which was drastically reduced in U373 cells exposed to compound 1 (Figure 3B). In contrast, this reduction of Cdc37 Ser13 phosphorylation was not observed with 50 μM TBB or compound 23.

### Structure Activity Relationship of azonaphthalene compounds on CK2α

To define the features that confer inhibitory potency to compound 1, we tested several derivatives *in vitro* and in the context of a complex cellular milieu (Table 1 and Table S1). First, compounds showing a CK2 inhibiting activity *in vitro* were tested at increasing concentrations for 24 or 48 h in the cellular CK2 assay. Five compounds inhibited CK2 in this assay: 1, 3, 4, 5 and 6 (Figure 3A and 3B, Table 1B). The most potent compounds were: 1 > 4, 5 >3 > 6.

**Table 1:**
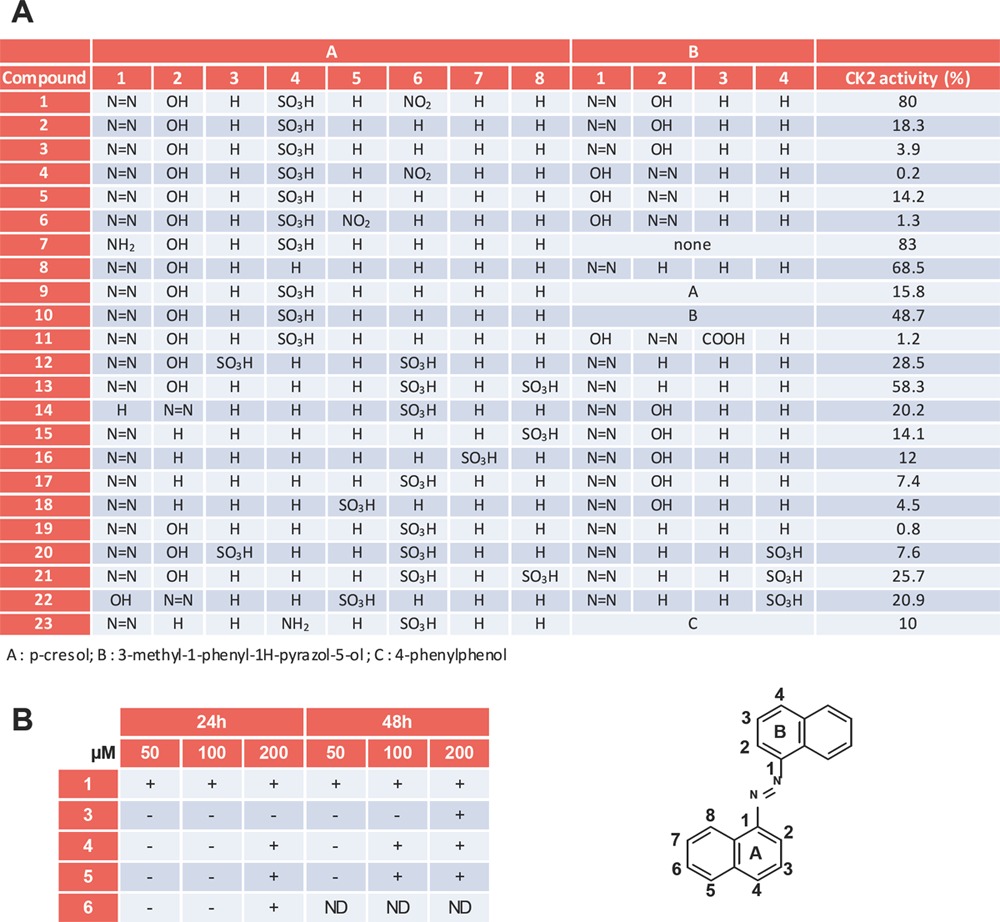
Features that confer inhibitory potency to compound 1 **A.** CK2 kinase assays were performed in the absence or presence of 10 μM compound. Results are expressed as a percentage of the control activity without inhibitor. **B.** Compounds that are cell permeable CK2 inhibitors. CK2 inhibition was monitored as in

Given that this class of compound possess a symetrical scaffold made of two naphthyl parts (A and B) bound by an azo function, we first investigated the requirement of part A. Compound 6, which possesses a nitro function at position A5 was fully active both *in vitro* and in the CK2 cellular assay. Moreover, compound 2 which lacks nitro function at position A6 remains active *in vitro* and compound 3 (which is zinc complex of compound 2) remains active in our cellular assay. Since nitro function is not necessary, it will be possible to remove it thereby avoiding possible carcinogenic metabolite. To gain insight into the requirements of the hydroxyl function in position A2 and the sulfonic acid in position A4, several derivatives of compound 1 were tested. Compound 8 which lacks the nitro, hydroxyl and sulfonic acid functions was notably inactive. This indicates a clear requirement for hydroxyl and/or sulfonic acid functions. Position variations of sulfonic acid function (compounds 14-18) lead to *in vitro* active compounds, indicating a requirement for sulfonic acid function. However none of these compounds are active in the CK2 cellular assay. Thus, hydroxyl function at position A2 and sulfonic acid at position A4 is, up to now, the optimal combination to get cell-potent compounds. Moreover, both compounds 3 and 5 are active both *in vitro* and in a CK2 cellular assay, indicating some tolerance in the position of the azo linker.

We next investigated the requirements of part B. Removal of this moiety is detrimental (compound 7). This indicates that this part of the molecule possesses functions conferring activity both *in vitro* and in our CK2 cellular assay. Testing of compound 11 reveals that an additional carboxylic function enhances the *in vitro* activity. Compounds 9 and compound 10 were much less active. This suggests that replacement of the naphthalene core is also detrimental. Moreover compounds 9-11 are also inactive in the CK2 cellular assay showing that Naphth-2-ol is, so far, the sole substituent conferring activity in a cellular context.

Collectively, it appeared from this SAR analysis that compound 4 was the most potent inhibitor in this compound series, being 10-fold more powerful than compound 1. Therefore, further studies were focused on compound 4.

### Analysis of CK2 inhibition by compound 4

**Figure 4:**
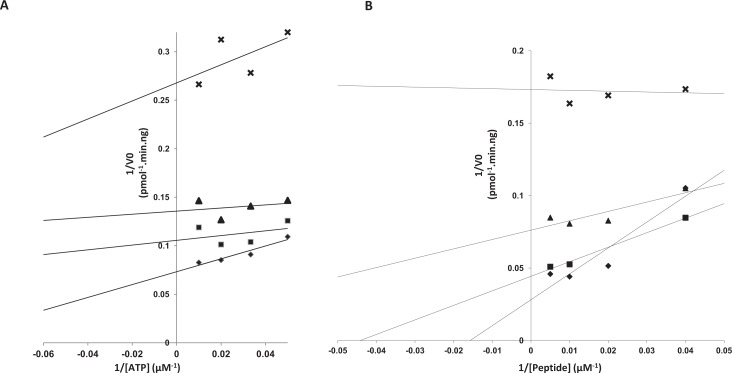
Characterization of compound 4 as a reversible non-competitive CK2 inhibitor. Lineweaver–Burk inhibition plots of human recombinant CK2α by compound 4 CK2 kinase activity was determined as described in the experimental section in the absence (♦) or in the presence of 0.2 (■), 0.4 (▲) and 0.8 (X) μM compound **4** with various concentration of ATP (**A**) or peptide substrate (**B**). The data represent means of experiments run in triplicate with SEM never exceeding 10%.

Dose-response curves for CK2 inhibition showed that compound 4 displayed an IC_50_~0.4 μM on CK2α (Figure S2). To define the mode of CK2 inhibition by compound 4, we performed steady-state kinetic analysis. It appeared that inhibition of CK2α by compound 4 was not competitive toward both ATP and peptide substrate (Figure 4). Again, plots were non linear with regards to peptide substrate concentrations. CK2 inhibition was strongly increased at high peptide substrate concentrations (Figure S3), a feature in line with the mode of inhibition of compound 1 (Figure 1D and S3).

### Selectivity profiling of compound 4

Specificity is a major concern for kinase inhibitor development. The kinase panel tested included members of major human protein kinase families [31] (Fig S4). This screening revealed that at 5μM, compound 4 inhibited CK2 by more than 95%, but had almost no effect on the other protein kinases tested (Table 2). Such high selectivity rules out compound 4 as promiscuous inhibitor. We calculated a previously described metric for kinase inhibitor selectivity based on the Gini coefficient [32]. The Gini score reflects, on a scale of 0 to 1, the degree to which the inhibitory activity of a compound (calculated as the sum of inhibition of all kinases) is directed toward only a single kinase (a Gini score of 1) or is distributed equally across all tested kinases (a Gini score of 0). Not surprisingly, the calculated Gini coefficient for compound 4 was 0.803 (Fig S5), a value among the highest for CK2 inhibitors [33, 34] and for kinase inhibitors in general [35]. Such conclusion is also supported by the “hit rates” i.e. the number of kinases inhibited by >50% by compound 4 using the 42 kinase panel: its value is 1, highlighting high specificity and suggesting that this compound might have characteristics of a uni-specific kinase inhibitor [35].

**Table 2:** Kinase Selectivity profile of compound 4 Residual kinase activity determined in the presence of 5 μM inhibitor is expressed as percentage of the control activity without inhibitor. Final concentration of ATP in the experiment was 100 μM.

Protein kinase	Residual activity (%)	Protein kinase	Residual activity (%)	Protein kinase	Residual activity (%)	Protein kinase	Residual activity (%)
**CK2α2(h)**	4	PKBα(h)	95	PDGFRα(h)	106	LOK(h)	114
**PKA(h)**	57	MKK7β(h)	96	ROCK-I(h)	106	CDK6/cyclinD3(h)	115
**Lyn(h)**	63	TAK1(h)	96	CDK1/cyclinB(h)	109	CHK1(h)	121
**AMPKα1(h)**	65	c-RAF(h)	97	MEK1(h)	110	p70S6K(h)	122
**CK1γ1(h)**	82	Pim-1(h)	97	PAK2(h)	110	ASK1(h)	124
**MST1(h)**	88	eEF-2K(h)	98	CaMKI(h)	111	PKCθ(h)	128
**NEK11(h)**	89	EGFR(h)	98	mTOR(h)	111	CDK7/cyclinH/MAT1(h)	129
**Plk3(h)**	90	PKCα(h)	100	MKK6(h)	112	IRAK4(h)	145
**DRAK1(h)**	94	CDK2/cyclinA(h)	101	MLK1(h)	112	ALK(h)	148
**JAK2(h)**	94	EphA5(h)	101	KDR(h)	113		
**Fyn(h)**	95	Abl(h)	103	IKKα(h)	114		

### Analysis of compound 4 binding site

**Figure 5:**
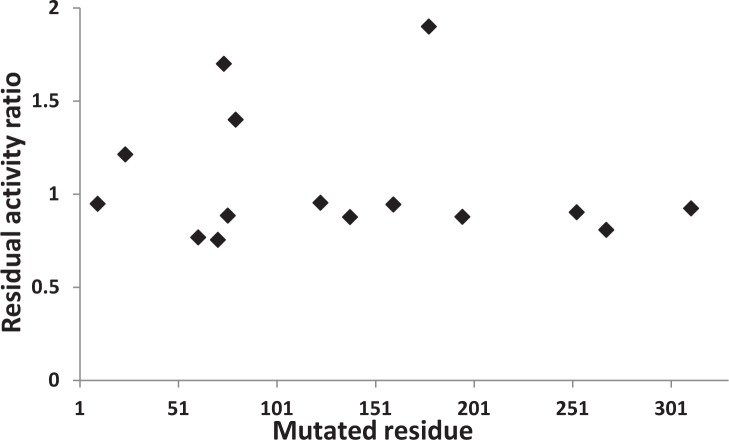
Compound 4 shares overlapping binding site with compound 1 Inhibitory effect of compound 4 on WT and a panel of mutants with the following residues mutated to alanine (10, 24, 61, 71, 74, 76, 80, 123, 138, 160, 178, 195, 253, 268, 308, 311). Residual activity ratio is defined as the normalized activity of mutant CK2α divided by the normalized activity of wild-type CK2α in presence of 0.4μM compound 4. Normalized activity represents the percentage of residual activity in presence of 0.4 μM compound 4.

Despite several attempts we could not get diffracting crystals to solve the 3D structure of a CK2-4 complex. Therefore, to delineate a potential binding site of compound 4, we tested its inhibition on a series of CK2α mutants (Figure 5). Strikingly, this revealed that CK2 mutants that were found insensitive to compound 1, were also resistant to compound 4 inhibition. This strongly predicts that both compounds have a common binding site located on helix αC and on the activation segment of CK2α.

Taken together this data indicates that compound 4 and compound 1 have a common, or at least overlapping binding site and inhibit CK2 in a similar manner.

### Compound 1 and 4 promotes cell cycle arrest

**Figure 6:**
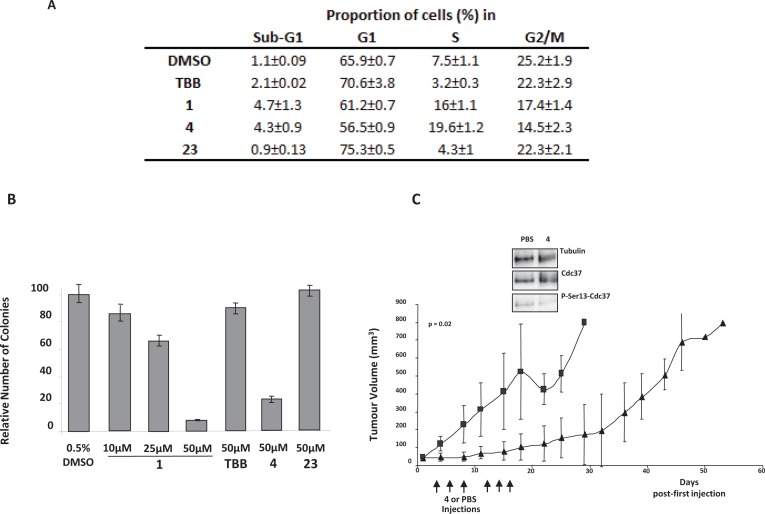
Azonaphthalene derivatives promote cell cycle arrest, reduce colony formation and exert anti-tumoral effect in vivo Nearly confluent U373 cells were treated with compounds or equivalent amount of DMSO. After 48h, cells were harvested and labeled with propidium iodide. DNA content was analysed with FACScalibur and Cell Quest software. Experiment was repeated three times. Proportion of U373 cells in sub-G1, G1, S and G2/M phases are summarized. **B.** Colony formation assay by soft agar culture. U373 cells were poured on an agarose layer (0.6%) mixed with agarose (0.3%) containing compounds for colony formation assay (soft agar). Fifteen days later, colonies (more than 20 cells) were counted in 10 fields/well. Experiment was performed in duplicate at least three times. **C.** Athymic nude mice were inoculated subcutaneously into the right flank with 7.5×10^5^ U373 cells. When tumor reaches ± 50 mm^3^, animals were treated intratumorously 3 times weekly for 2 weeks, with compound **4** dissolved in PBS (1 mg/20 μl/injection) or PBS (control group). Tumor volume was determined twice weekly. Results represent the average of the tumor volume of 5 mice per group. Insert: representative western blots. Tumor extracts were analyzed by western blot for expression of tubulin, Cdc37 and P-Ser13-cdc37 [29].

We then focus our attention on the U373 cell line because they were particularly responsive to compound 1. These cells originate from glioblastoma, an aggressive tumor of the brain with poor survival prognostic. They harbor a p53 mutant isoform and are particularly resistant to drug-induced apoptosis [36]. First, we explored whether compound 1 could inhibit their cell cycle as it was already observed for compound 4 in endothelial cells [37] and A431 epidermoid carcinoma cells [38]. Cells were treated with 50 μM of compound 1 for 48h or equivalent concentration of TBB, compounds 23, 4 or DMSO and then labeled with propidium iodide. U373 cells exposed to compounds 1 or 4 exhibited a cell cycle arrest with a substantial accumulation in mid-S-phase (Figure 6A).

### Azonaphthalene derivatives inhibit colony formation and tumor growth

To assert the potential clinical interest of azonaphtalene derivatives, we first tested their ability to inhibit colony formation using soft agar assay as an *in vitro* surrogate of tumorigenesis. U373 cells can grow without anchorage and form colonies in soft agar culture reflecting their malignant properties. Colony formation assays were performed using compounds 1, 4, and compound 23 and TBB as controls. Both Compound 1 and 4 inhibited colony formation in a dose dependent manner while TBB or compound 23 were without effect at the same concentration (Figure 6B).

The NCI DTP website shows that compounds 1 and 4 that were the most active in a colony formation assay, have a good pharmacological profile (http://dtp.nci.nih.gov/docs/invivo/invivoscreen.html) a property which was confirmed by the group of Atassi [38]. Compounds 3, 5 and 6 were not evaluated by the NCI.

We next tested the azonaphthalene compounds in a murine U373 tumor regression assay. We chose compound 4 for further *in vivo* investigations because of its high potency and specificity. The data illustrated in Figure 6C show that intratumoral injection of compound 4 could decrease tumor incidence across all time points compared to mice injected with PBS. After 3 weeks, mice injected with compound 4 had tumors that were six times smaller than those injected with PBS (Figure 6C). Western blot analysis showed that Cdc37 Ser13 phosphorylation in U373 tumors, was significantly reduced in mice injected with compound 4 compared to mice injected with PBS (Figure 6C).

To assess whether the anti-tumoral effect was related to CK2 inhibition, we wanted to inject mice with compound 23 that was used as inactive control in colony formation assay and CK2 activity cellular assay. However, a previous study [30] has shown that this compound displays antitumoral effect via inhibition of neoangiogenesis. Therefore, mice were treated with compound 9 (Calmagite) which was also inactive in the CK2 activity cellular assay. No significant anti-tumoral effect could be observed between mice treated with compound 9 and control vehicle (Figure S6). Collectively, these observations suggest that tumor growth inhibition by compound 4 is likely related to CK2 inhibition.

## DISCUSSION

Abnormally high CK2 expression provides cancer cells with an environment favorable for tumor development [39]. Therefore development of CK2 inhibitors may represent an interesting approach for the treatment of cancer. Most CK2 inhibitors are ATP-competitive but new development in the kinase field has raised interest in exploiting alternative mode of inhibition [40]. In this work, we describe a family of chemicals that are non-ATP competitive and cell potent CK2 inhibitors with good profile for their *in vivo* use.

The exquisite selectivity of compound 4 revealed by its high Gini coefficient, could reflect unique features of CK2 and be related to the allosteric mechanism of action of this inhibitor. Although strong kinase selectivity may not be essential for efficacy of therapeutic agents, it is critical for tool compounds used to elucidate kinase biology.

Site-specific mutations of Lys74Ala, Arg80Ala or Leu178A in CK2α showed that these mutant forms exhibit some resistance toward compound 1 and 4 inhibition. Interestingly, these three mutations are located in or close to structural elements that are crucial for CK2 activity, namely the substrate binding site, the RD and DWG motifs [41, 42]. By interfering with these regulatory elements, compound 1 and 4 may stabilize a non-productive catalytic form of CK2α.

We consistently observed parabolic inhibition plots in the presence of increasing peptide substrate concentrations, a feature that may arise from allosteric effects. In this scenario, a substrate-dependent “conformation selection” would favor inhibitor binding.

Our SAXS analysis showed that the overall shape of a CK2α-compound 1 complex is different from active CK2α shape obtained either by SAXS (this work) or by X-ray crystallography [43]. Moreover, the shape of the CK2α-compound 1 complex is also different from a structure of an inactive CK2α conformation [22]. Taken together, the binding of compound 1 may distort critical regulatory elements and lock the kinase in a new non-productive conformation. Further structural analysis will be required to precisely reveal the binding site of these inhibitors.

Azonaphthalene derivatives have been described as nonpeptidyl thrombopoietin mimics [44]. We show that molecules belonging to this family of chemicals are cell-permeable and can target the cellular CK2 activity. A panel of tumor cell lines exposed to compound 1 showed a decreased viability that was correlated with a down regulation of CK2 activity. Indeed, involvement of CK2 in cell cycle regulation is well documented. CK2 is required at multiple transitions in the cell cycle (including G0/G1, G1/S and G2/M). We found that U373 cells exposed to compounds 1 or 4 exhibited a cell cycle arrest with a substantial accumulation in mid-S-phase. These observations are in accordance with similar effects of compound 4 previously described in endothelial cells [37]. Whether activation of apoptosis that occurs in HeLa cells treated with high doses of compound 1, is a consequence of a cell cycle arrest or direct promotion of apoptosis remains to be established. Of note, the viability of U373 cells which are particularly resistant to drug-induced apoptosis [36] was strongly affected by compound 1 and this effect was correlated with a down regulation of CK2 activity.

Compound 1 inhibited U373 cell colony formation in a dose dependent manner. Of note, CK2 phosphorylation of HSP90 modulates chaperone function and drug sensitivity [45]. By promoting activities of several oncokinases, the Cdc37/Hsp90 chaperone complex contributes to the acceleration of cell proliferation observed in cancer cells. Like CK2, Cdc37 is over-expressed in cancer cells and a target for cancer therapy [46]. To interact properly with client kinases, Cdc37 must be phosphorylated on Ser13 by CK2. We showed that compound 1 decreases Cdc37 phosphorylation on Ser13 both in U373 cells and in tumor-bearing mice. We can hypothesize that at least part of the anti tumorigenic effect of this class of compound could involve inhibition of Cdc37 phosphorylation. Interestingly, it was recently reported that Sarcoma 180 tumors were sensitive to compound 1 showing a ~50% tumor regression in treated mice (NCI DTP website). Cdc37 Ser13 phosphorylation in glioblastoma tumors was also significantly reduced in mice injected with compound 4 suggesting that at least part of the antitumoral effect of this class of inhibitors could involve inhibition of Cdc37. We cannot exclude that the effect of azonaphthalene derivatives could be also explained by the partial inhibition of other targets. However, to date compound 4 is among the most selective CK2 inhibitors published.

In this work, we showed that compound 4 induced a significant inhibition of glioblastoma tumor growth that was correlated with CK2 inhibition. Of note, compound 4 was reported to inhibit angiogenesis in a chick chorioallantoic membrane assay and proliferation of primary endothelial cells, A431, L1210 and M5076 cancer cells [38]. In this study, the authors suggested that the effect of compound 4 could involve cell cycle perturbation through inhibition of topoisomerase II catalytic activity [38]. Interestingly, topoisomerase II is a known CK2 target [47].

In conclusion, we described the discovery of a family of Azonaphthalene derivatives that perturb CK2α conformation thereby blocking productive binding of substrates. These compounds may be useful as research tools in probing CK2 conformational plasticity because our kinetic analysis showed a mechanism of action consistent with an allosteric mode of inhibition, highlighting the opportunity of exploiting different CK2 inhibition mechanisms [19]. Although further structural analysis will be required to reveal the binding site of these inhibitors, their therapeutic potential is emphasized by their efficacy in growth inhibition of apoptosis-resistant tumors. Further optimization of these compounds and the results from further *in vivo* testing may aid in the design of other classes of CK2 inhibitors and should reveal their efficacy in additional specific models.

## METHODS

### Cell culture

HeLa (cervical adenocarcinoma), U373 (glioblastoma) and MDA231 (breast adenocarcinoma) cell lines were cultivated in Dulbecco's medium (Invitrogen) Life Technologies, Inc.) while A549 (lung carcinoma), H1299 (lung carcinoma) and LnCap (prostate carcinoma) cell lines were cultivated in RPMI (Invitrogen) Life Technologies, Inc.). Each medium was supplemented with 10% (v/v) fetal calf serum (FBS, BioWest). MCF7 (breast adenocarcinoma) cell line was cultivated in Dulbecco's medium with insulin (10μg/ml).

### High-throughput screening

High-throughput screening of the NCI Diversity Set chemical library (2,860 compounds) was performed as previously described [48]. Active fractions from the primary screen were retested from freshly made solutions.

### Cellular CK2 activity assay

A cellular CK2 activity assay was performed as previously described [28].

### Western Blot

Western-blotting was performed as previously described [11] using the following antibodies: anti-CK2α antibody [3] at 1/5000, or anti-Ser13-phospho Cdc37 [29] at 1/3000, anti-Cdc37 (Santa Cruz, sc13129) at 1/2000 and anti-tubulin (YL1/2, Abcam) at 1/50000.

### Kinase selectivity profiling

Kinase selectivity of compound 4 was assessed using the Kinase Profiler service offered by Millipore which utilizes a radiometric filter-binding assay. The assays were performed at 100 μM ATP in the presence of 5 μM inhibitor. Inhibition, expressed as the percent of activity determined in the absence of inhibitor, was calculated from the residual activity measured in the presence of 5 μM inhibitor.

### Selectivity parameters

Lorenz curves were derived from the selectivity data. Gini coefficients and Hit rates were calculated as described in [32].

### Cell Viability

Cell viability assay was performed as described previously [28]

### Cell cycle distribution analysis

Cell cycle distribution analysis was performed as described previously [15].

### Soft agar assay

Soft agar assay was performed as described previously [15].

### CK2 Phosphorylation assays

CK2 radiometric kinase assay based on conventional filter-binding assay was performed as previously described [20].

### CK2α production and purification

GST-rhCK2α^∆C^(1-335) expression in *E.Coli* BL21 cells after induction with IPTG and lysis were performed as described in [15]. Supernatant was added onto glutathion-sepharose beads (Amersham). After overnight incubation, beads were washed by 40mM Tris pH 7.5, 150 mM NaCl and cleavage reaction was performed on column (1 unit of thrombin.mL^−1^ of beads (Sigma, T6634) in PBS containing 200 mM NaCl, 2% glycerol and 1 mM DTT for 4h at 4°C). Following elution (50 mM Tris pH 7.5, 150 mM NaCl) the proteins were loaded on a heparine-sepharose column equilibrated in the elution buffer. Proteins were then eluted with a linear gradient of 0.15-1M NaCl in 50 mM Tris pH 7.5. The active fractions were pooled and concentrated with a Centricon Plus-70mL (Millipore). Production and purification of GST-rhCK2α mutants were carried out using the same protocol.

### SAXS experiments

SAXS experiments were performed at the SOLEIL Synchrotron (Saclay, France) on beamline SWING. The wavelength λ was 1.0 Å (1 Å=0.1 nm) and the sample-to-detector distance was 1924 mm giving access to scattering vectors q ranging from 0.009 to 0.47 Å-1.The scattering vector is defined as q = 4π /λ sinθ, where 2θ is the scattering angle. The detector was an AVIEX170170 CCD detector, and 40 successive frames of 4.2 ms exposure were recorded for each sample. The samples were injected with an automatic sample-changer and circulated through an evacuated quartz capillary between each frame to avoid radiation damage.

Protein concentration of CK2α (in 25 mM Tris pH 8.5, 0.2 M NaCl, 1 mM DTT and 10% glycerol) was varied from 1.25 to 10 mg⋅ml^−1^ in order to check for interparticle interactions. The protein concentration of CK2α in the presence of 500 μM compound 1 (CK2α/1 ratio 1:2) was varied from 1.25 to 10 mg⋅ml^−1^. The radius of gyration *R*_G_ was derived by the Guinier approximation *I*(*q*)=*I*(*0*)⋅exp(*−q*^2^*R*_G_^2^/3) for *qR*_G_<1.0. The distance distribution function *P*(*r*) was calculated by the Fourier inversion of the scattering intensity *I*(*q*) using GNOM [49]. The maximum diameter of the macromolecule, *D*_max_, was also determined [50].

Using the program GASBOR [51], the overall shapes of CK2α alone or in complex with compound 1 were restored from the experimental data. Ten independent fits were run with no symmetry restriction, and the stability of the solution was checked. The atomic structure of CK2α (PDB ID 1PJK) was then fitted in the averaged calculated shape, using SUPCOMB [52]. The theoretical scattering curve of CK2α (PDB ID 1PJK) was calculated using CRYSOL [53].

### Murine xenograft tumor growth assays

All experimental procedures adhered to our local ethical committee (Comité régional d'éthique pour l'Expérimentation animale CREEA, Rhône Alpes - protocol n°315). Female Harlan athymic nude mice (6-8 weeks) were inoculated subcutaneously into the right flank with 7.5×10^5^ U373 cells. When tumors reaches ± 50 mm^3^ (volume = length × width × height), animals were treated intratumorously 3 times weekly for 2 weeks, with compound 4 dissolved in PBS (1 mg/20 μl/injection) or PBS (control group). Body weight and tumor volume were determined twice weekly. The experiment was terminated when tumor volume was about 1000 mm^3^. Data are expressed as means and s.d. and were analysed with Student's *t*-test; significance is defined as *p*<0.05.

Tumor extracts were prepared in RIPA buffer and analyzed by western blotting.

## Supplementary Figures and Tables





## ABBREVIATIONS

CK2: Casein Kinase 2
SAXS: Small Angle X-ray Scattering
TBB: 4,5,6,7-TetraBromo-1H-Benzotriazole
R_G_: radius of gyration
