# Supplementary material for: Antitumoral activity of allosteric inhibitors of protein kinase CK2
Source: Oncotarget. 2011 Dec 14;2(12):997–1010. doi: 10.18632/oncotarget.361 (PMC3282105; doi:10.18632/oncotarget.361)
Supplement: Supplementary file 1 [file oncotarget-02-997-s001.pdf]

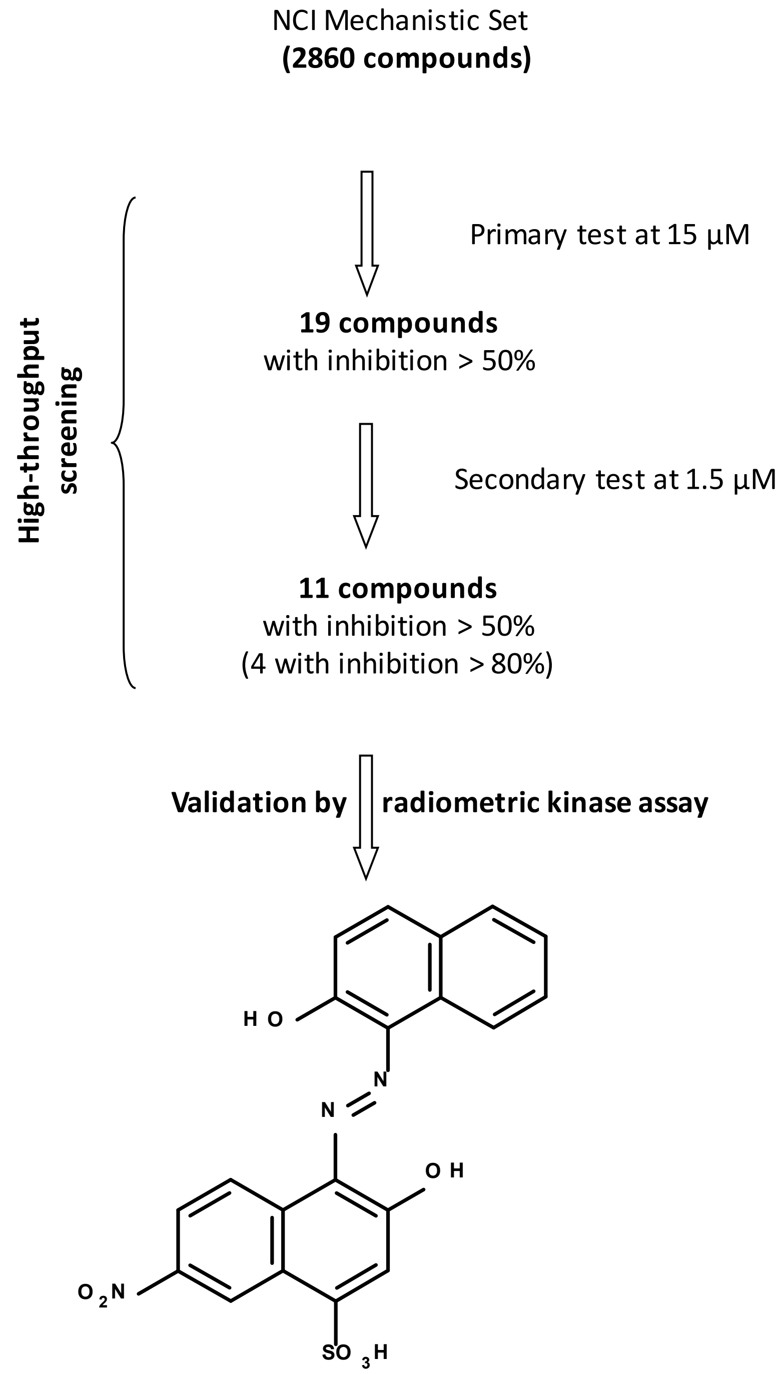

Compound 1

Figure S1. Identification of compound 1 as a CK2 inhibitor.
Flow-chart of the automated screening of chemical libraries for the identification of diazostilbene derivatives as CK2α inhibitors.

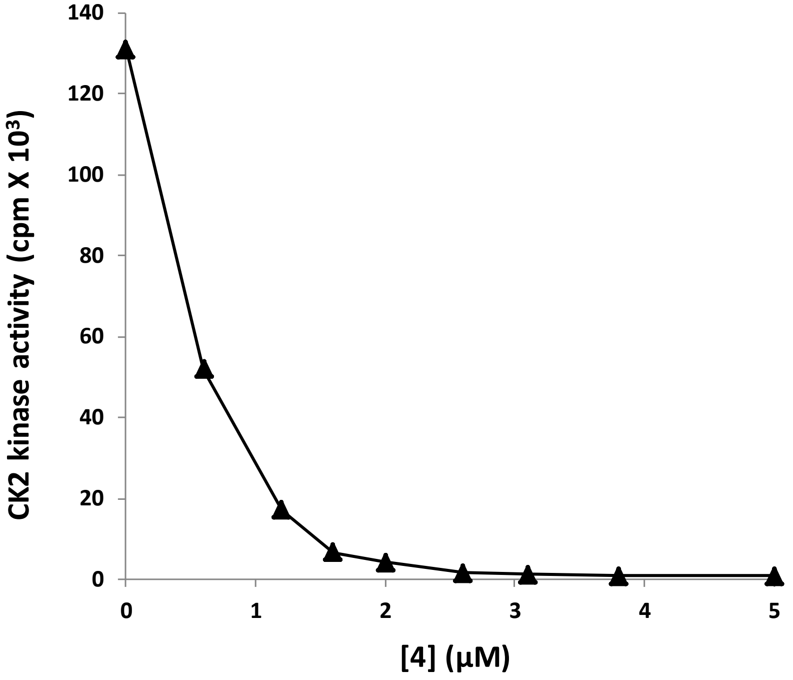

Figure S2. Inhibition of CK2α by compound 4.
Inhibition of the catalytic activity of CK2α by increasing concentrations of compound **4**.

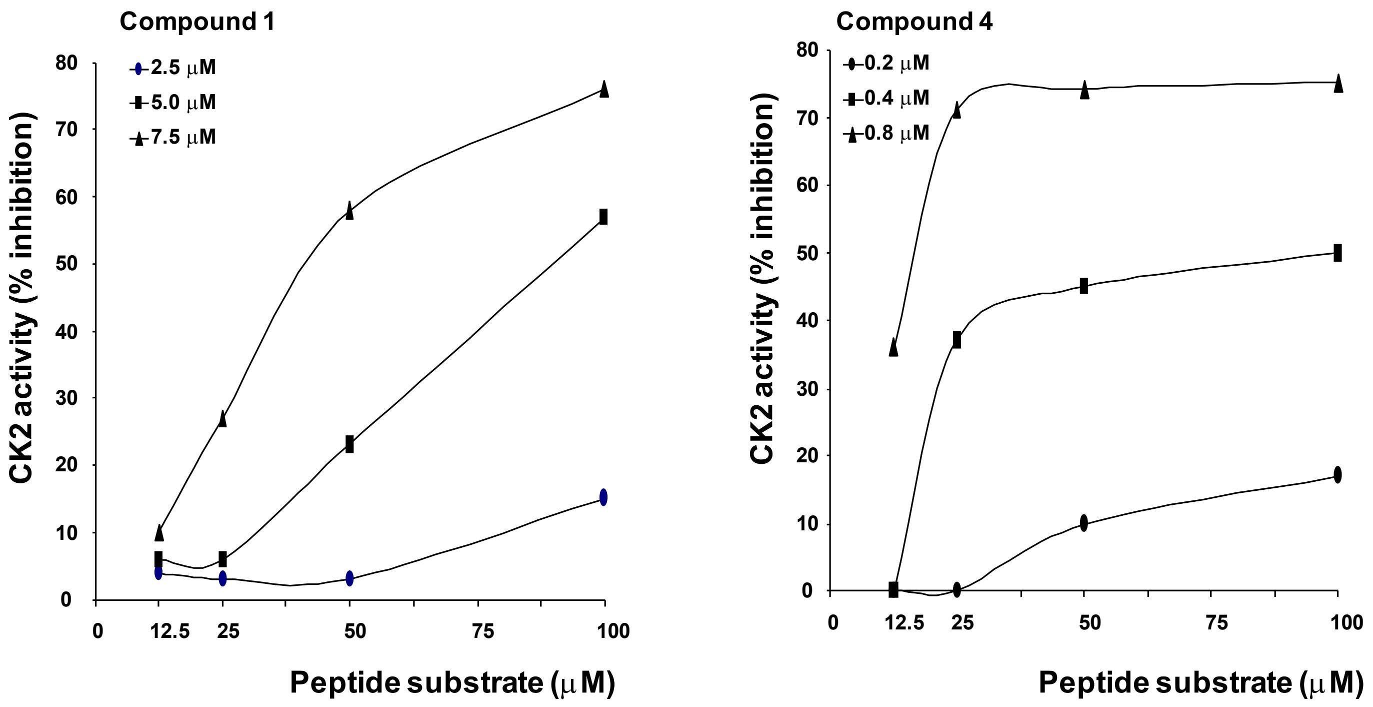

Figure S3. Substrate-dependent CK2 inhibition by compound 1 and 4.
CK2 kinase activity was determined as described in the experimental section in the absence or in the presence of increasing concentrations of compound **1** (left panel) or compound **4** (right panel) with various concentrations of peptide substrate.
The data represent means of experiments run in triplicate with SEM never exceeding 10%.

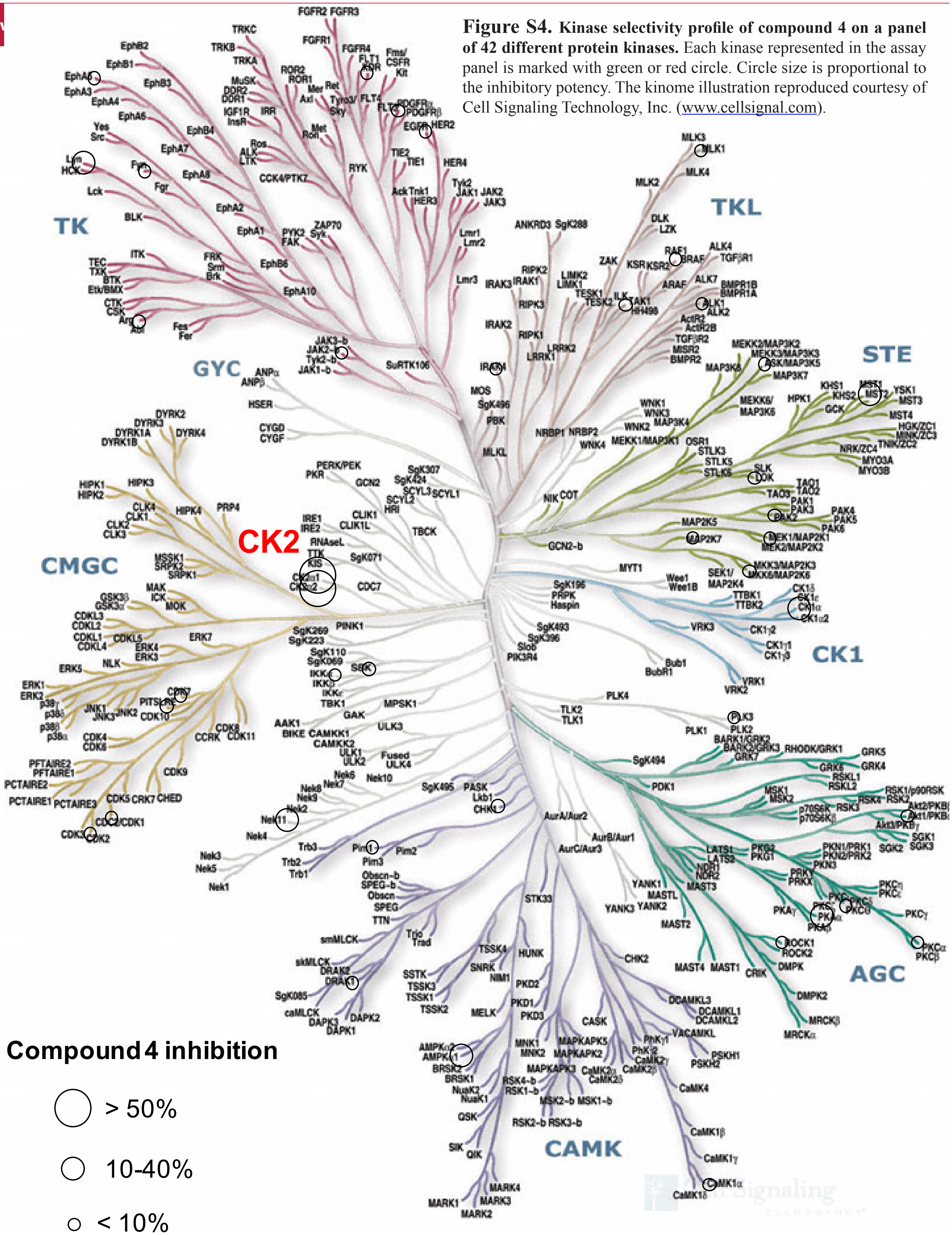

Figure S4. Kinase selectivity profile of compound 4 on a panel of 42 different protein kinases. Each kinase represented in the assay panel is marked with green or red circle. Circle size is proportional to the inhibitory potency. The kinome illustration reproduced courtesy of Cell Signaling Technology, Inc. (www.cellsignal.com).

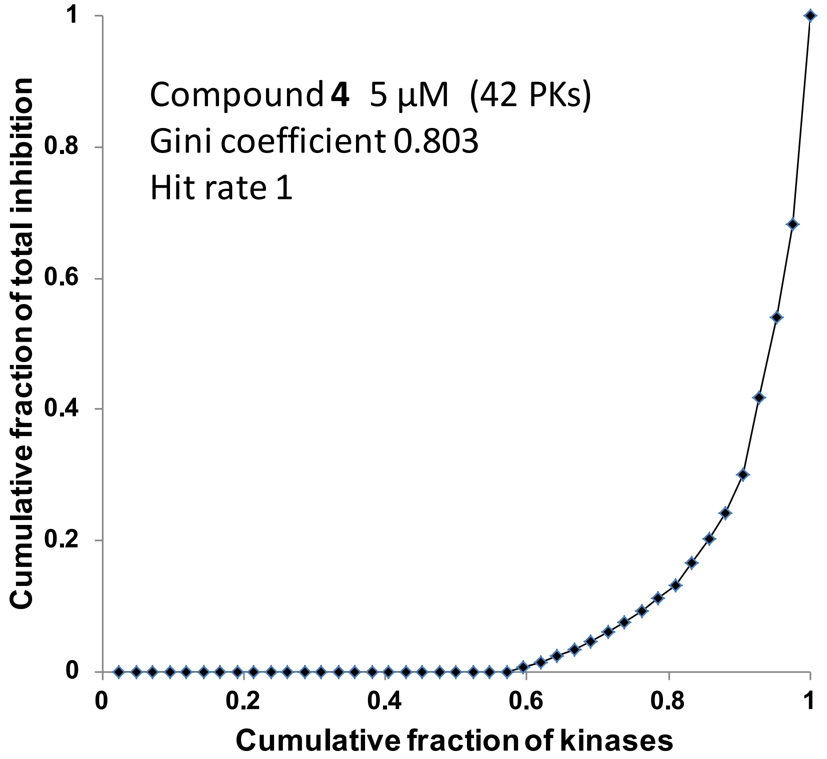

Figure S5. Lorenz curves, Gini coefficients and Hit Rates for compound 4.
For details see the experimental procedures.

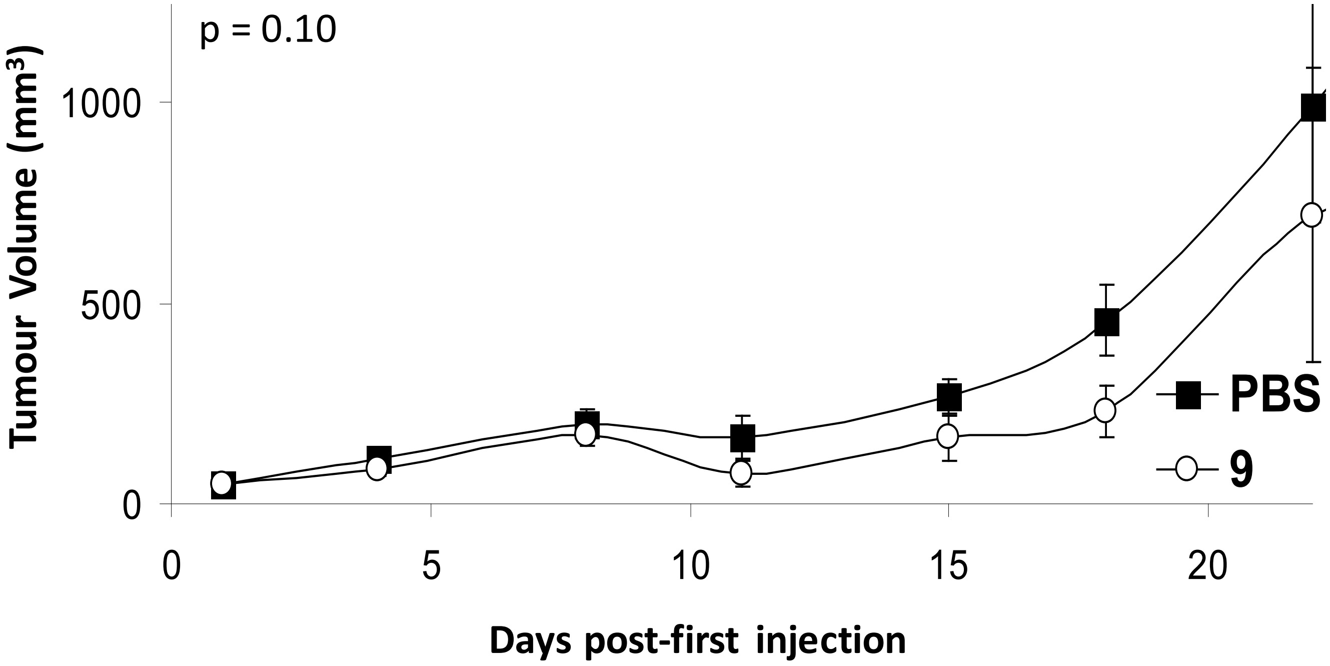

Figure S6. Lack of in vivo antitumoral effect of compound 9.
Athymic nude mice were inoculated subcutaneously into the right flank with 7.5x10^5 U373 cells. When tumor reaches ± 50 mm^3, animal was treated intratumorously 3 times weekly for 2 weeks, with compound **9** dissolved in PBS (1 mg/20 µl/injection) or PBS (control group). Tumor volume was determined twice weekly. Results represent the average of the tumor volume of 5 mice per group.
