# Supplementary material for: Antitumoral activity of allosteric inhibitors of protein kinase CK2
Source: Oncotarget. 2011 Dec 14;2(12):997–1010. doi: 10.18632/oncotarget.361 (PMC3282105; doi:10.18632/oncotarget.361)
Supplement: Supplementary file 2 [file oncotarget-02-997-s002.doc]

**Table S1.** **Azonaphthalene derivatives (compounds 1 to 23**)**.**

**Compounds used in this work.**

|  |  |  |  |  |  |
| --- | --- | --- | --- | --- | --- |
|  | **Compound** | **CAS N°** | **Name** | **Supplier** |  |
|  |  |
|  | **1** | 3618-58-4 | NSC73413 | NCI |  |
|  | **2** | 2538-85-4 | Calcon | Sigma |  |
|  | **3** | 5410-93-5 | NSC4203 | NCI |  |
|  | **4** | 1787-61-7 | Eriochrome Black T | Sigma |  |
|  | **5** | 3564-14-5 | Eriochrome Blue Black B | Sigma |  |
|  | **6** | 518-88-7 | NSC85561 | NCI |  |
|  | **7** | 116-63-2 | 4A3H1NSA | Sigma |  |
|  | **8** | 2653-64-7 | NSC45575 | NCI |  |
|  | **9** | 3147-14-6 | Calmagite | Sigma |  |
|  | **10** | 3618-63-1 | Eriochrome Red B | Sigma |  |
|  | **11** | 3737-95-9 | Calconcarboxylic acid | Sigma |  |
|  | **12** | 5858-33-3 | NSC45583 | NCI |  |
|  | **13** | 2766-77-0 | NSC45582 | NCI |  |
|  | **14** | 5850-95-3 | NSC45579 | NCI |  |
|  | **15** | 6300-38-5 | NSC45578 | NCI |  |
|  | **16** | 85136-41-0 | NSC45577 | NCI |  |
|  | **17** | 68133-05-1 | NSC45576 | NCI |  |
|  | **18** | 5850-93-1 | NSC45205 | NCI |  |
|  | **19** | 5864-86-8 | NSC45206 | NCI |  |
|  | **20** | 915-67-3 | NSC215207 | NCI |  |
|  | **21** | 2611-82-7 | NSC4301 | NCI |  |
|  | **22** | 3756-79-0 | NSC47716 | NCI |  |
|  | **23** | 6946-03-7 | NSC65828 | NCI |  |
|  |  |  |  |  |  |
